# Consistently Showing Your Best Side? Intra-individual Consistency in #Selfie Pose Orientation

**DOI:** 10.3389/fpsyg.2017.00246

**Published:** 2017-02-21

**Authors:** Annukka K. Lindell

**Affiliations:** Department of Psychology and Counselling, School of Psychology and Public Health, La Trobe University, BundooraVIC, Australia

**Keywords:** left, right, emotion, photo, selfie, self-portrait

## Abstract

Painted and photographic portraits of others show an asymmetric bias: people favor their left cheek. Both experimental and database studies confirm that the left cheek bias extends to selfies. To date all such selfie studies have been cross-sectional; whether individual selfie-takers tend to consistently favor the same pose orientation, or switch between multiple poses, remains to be determined. The present study thus examined intra-individual consistency in selfie pose orientations. Two hundred selfie-taking participants (100 male and 100 female) were identified by searching #selfie on Instagram. The most recent 10 single-subject selfies for the each of the participants were selected and coded for type of selfie (normal; mirror) and pose orientation (left, midline, right), resulting in a sample of 2000 selfies. Results indicated that selfie-takers do tend to consistently adopt a preferred pose orientation (α = 0.72), with more participants showing an overall left cheek bias (41%) than would be expected by chance (overall right cheek bias = 31.5%; overall midline bias = 19.5%; no overall bias = 8%). Logistic regression modellng, controlling for the repeated measure of participant identity, indicated that sex did not affect pose orientation. However, selfie type proved a significant predictor when comparing left and right cheek poses, with a stronger left cheek bias for mirror than normal selfies. Overall, these novel findings indicate that selfie-takers show intra-individual consistency in pose orientation, and in addition, replicate the previously reported left cheek bias for selfies and other types of portrait, confirming that the left cheek bias also presents within individuals’ selfie corpora.

## Introduction

Selfies (digital self-portrait photographs taken with a smartphone or webcam) are now ubiquitous. Whilst self-portraiture in art has a long history, its prevalence as a vernacular photographic genre is novel (Walker, 2014; Marwick, 2015), and growing: use of the word “selfie” increased over 17,000% between 2012 and 2013 (Oxford Dictionaries, 2013). Though selfies are undoubtedly about the self, “… they long for – require even – sharing to be considered “true” selfies” (Hess, 2015, p. 1631), thus selfies are uploaded to social media like Facebook and Instagram. This public sharing of selfies is very much the norm, with 17- to 47-year-old Polish participants posting up to 650 selfies per month on social media sites (average 14.01 posted per month for females; 7.62 posted per month for males; Sorokowska et al., 2016).

The selfie phenomenon was catalyzed by the advent of the digital smartphone. Because the smartphone’s front-facing camera provides a means for beholding oneself as the image is recorded (Walker, 2014; Frosh, 2015), people can exert a much higher degree of control over the way they self-represent than previously available. The fact that digital cameras provide photographs immediately, with no costs for film, means that selfies can be taken repeatedly, with the best carefully chosen, edited, and then uploaded to social media. The pose depicted is thus far from accidental (Lindell, 2017); every selfie available for public view on social media was first consciously selected and approved by the selfie-taker (Saltz, 2014). Although there is currently no academic research quantifying selfie-taking behavior, a recent market research survey conducted by OnePoll (*N* = 2000) found that females aged 16–25 spend over 5 h a week (48 min per day) taking selfies, with an average of seven selfies being taken for each ‘perfect selfie’ uploaded to social media sites (Strick, 2015). Not surprisingly then, selfie-takers perceive themselves as more attractive and likable in their selfies than in photographs taken by other people (Re et al., 2016).

As creating and sharing a selfie can be conceived as “an act of self-representation,” (Walker, 2014, p. 12), posing orientation in selfies has been examined to determine how selfie-takers self-represent. Such investigation is motivated by previous research that has established posing asymmetries in painted and photographic portraits: people are more likely to adopt a left than right cheek pose (LaBar, 1973; McManus and Humphrey, 1973). A number of different theories have been put forward to account for the left cheek bias. For example, accounts based on the effect of reading and writing direction suggest that readers of left-to-right languages show a left cheek bias, whereas readers of right-to-left languages instead show a right cheek bias (e.g., Pérez-González, 2012). The Spatial Agency Bias offers an alternate theory, suggesting that figures’ roles in artworks, whether as agents or receivers of action, guide their pose orientation. According to this account, passive portrait poses favor left cheek poses because they emphasize the absence of agency (Suitner and McManus, 2011). Recent analysis of moving images appears consistent with this proposal, finding that the lead male actors in action films do not show a left cheek bias (Bode et al., 2016). This finding is also consistent with the emotion-based account of the left cheek bias favored by the present study. Because the left side of the face is predominantly controlled by the emotion-dominant right hemisphere (Patten, 1996; Demaree et al., 2005), the left cheek is more emotionally expressive (Nicholls et al., 2004). Consequently, people intuitively offer the left cheek when asked to pose for a photo expressing emotion, and the right cheek when posing for a photo that conceals emotion (Nicholls et al., 1999). Consistently, viewers perceive models in left cheek poses as more emotionally expressive and open than identical models in right cheek poses (Nicholls et al., 2002; see Lindell, 2013b, for review).

Research confirms that the left cheek bias extends to selfies. Bruno and Bertamini (2013) first investigated selfie posing biases experimentally, finding that over 45% of participants (predominantly university student sample) adopted a left cheek pose when asked to take a selfie using an iPhone’s front-facing camera (33% right cheek pose; 23% midline frontal pose), with proportions consistent across genders. Subsequent selfie-taking investigations in schoolchildren (aged 9–16 years), and a community sample of adults, similarly confirmed the left cheek bias for both male and female single-subject selfies (Bruno et al., 2014, in press). Though Lindell (2017) reported a midline (49.8%), rather than left cheek (26.5%), posing bias across genders in a general population adult sample, the discrepancy from previous investigations is argued to reflect the conservative criterion for coding midline poses adopted [Lindell’s midline pose category encompasses three of Bruno and Bertamini’s (2013) pose categories: “slightly left,” “frontal,” “slightly right”; see Lindell (2017), for discussion]. Overall, the research suggests that like other types of portrait, selfie-takers favor left cheek poses.

Critically, the left cheek bias for selfies does not simply reflect a mechanical artifact: neither participant handedness nor the hand used to capture the image influences selfie pose orientation (Lindell, 2017). Instead, the left cheek bias observed for selfies appears consistent with that observed for painted (McManus and Humphrey, 1973) and photographic (LaBar, 1973) portraits of others, and is argued to reflect the sitters’ unconscious preference for displaying the more emotive left cheek (see Lindell, 2013b; Bruno et al., 2015, for discussion).

In keeping with experimental investigations of selfie-taking, examination of cheek biases in 3200 selfies uploaded to SelfieCity (an online selfie database, with images drawn from Instagram) also found a left cheek bias for standard selfies (selfies in which the selfie-taker points the camera toward themselves; Bruno et al., 2015). For mirror selfies, in which the selfie-taker poses in front of a mirror and takes a photo of their reflection, the posing bias reverses to a right cheek bias. As the mirror reverses left and right, a right cheek bias for mirror selfies indicates that the subjects adopted left cheek poses in front of the mirror, akin to the right cheek bias typically found in painted self-portraits (see Lindell, 2013a). Thus across selfie types, Bruno et al.’s (2015) SelfieCity study indicates that both male and female selfie-takers tend to pose offering their left cheek.

Previous investigations of posing orientation in selfies have all sampled a single selfie from multiple different participants (Bruno and Bertamini, 2013; Bruno et al., 2014, 2015, in press; Lindell, 2017). Whether the individual selfie-taker similarly shows a left cheek bias within his or her own corpus of selfies remains to be determined. Intra-individual investigation of selfie posing biases appears worthy of consideration because it illuminates the degree of consistency in posing orientation, determining whether selfie-takers repeatedly favor a preferred pose, or switch between the three pose types: left cheek, midline, and right cheek. Moreover, such investigation sheds light on the generalizability of the left cheek bias previously reported for selfies, based on samples comprised of multiple single selfies. The present study was thus designed to expand the selfie posing bias data by assessing intra-individual consistency in posing biases. An overall left cheek bias within an individual selfie-taker’s corpus would be in keeping with the left cheek bias previously observed across multiple subjects; an overall right cheek or midline bias, or a pattern in which the selfie-taker alternates evenly between the three posing options in their selfie corpus, would indicate that intra-individual patterns show a marked departure from those previously observed across individuals. Given that previous selfie investigations have found no difference in selfie posing biases between males and females (e.g., Bruno et al., 2015; Lindell, 2017), no gender effect was anticipated in the present investigation.

Selfies were sourced from Instagram: a free mobile application compatible with both iOS and Android operating systems. Instagram allows users to upload photographs (particularly selfies), manipulate them using filters, and share them with other people who may then comment on, and/or “like” the images (Marwick, 2015). There are over 500 million active Instagram users, uploading more than 95 million images per day^1^. Users can set their accounts to ‘public’ or ‘private’; only public accounts were included in the present investigation. At the time of writing over 275 million selfies had been uploaded to Instagram for public viewing using the hashtag “#selfie,” allowing the identification of images that users explicitly identified as selfies. Only single-subject selfies were sampled.

## Materials and Methods

### Selfie Sourcing

Selfies were sourced by searching Instagram using the #selfie. Single-subject selfies were identified, with the first 100 male and 100 female single-subject selfie uploaders selected as participants; the #selfie feed was refreshed to load more #selfie images until the full sample of male and female participants was collected. The most recent 10 single-subject selfies for the each of the 200 participants were then identified in each participant’s Instagram feed, resulting in a total sample of 2000 selfies. The duration of time over which participants uploaded 10 single-subject selfies to Instagram ranged from <1 day (two participants uploaded 10 single-subject selfies to Instagram in fewer than 24 h) to 590 days (*M* = 92.20 days; *SD* = 117.13 days).

### Selfie Coding

Each selfie was coded for the participant’s identity, selfie type (normal; mirror), selfie-taker’s sex (male, female), and pose orientation (left cheek, right cheek, midline). The coding criterion used to determine posing orientation was conservative: selfies that unambiguously presented one side of the selfie-taker’s face to the camera were classified “left” or “right.” In keeping with the method previously described by Bruno and Bertamini (2013) and Lindell (2017), selfies that depicted the subject in a pose that could not be immediately classified by eye were first enlarged to approximately 16.5 cm × 21 cm. The distances from the center tip of the subject’s nose to each side of his/her face were then measured to determine posing orientation: (left > right = left cheek pose; left < right = right cheek pose). Any difference of <2 mm was recorded as a midline pose (see Lindell, 2017).

## Results

**FIGURE 1:**
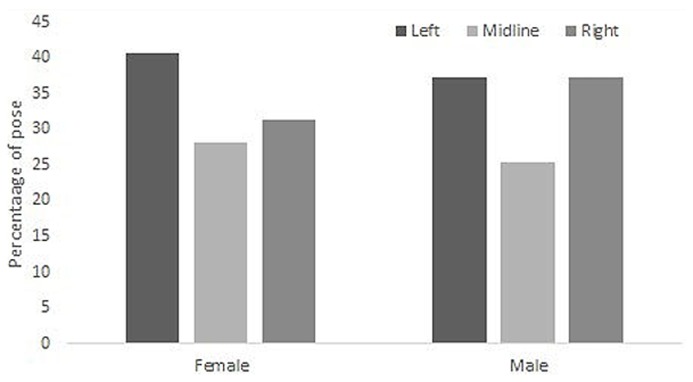
**Percentages of left, right, and midline selfie poses as a function of the selfie-takers’ sex (female, male).** Please note that ‘cheek’ refers to the anatomical cheek offered to the camera or mirror.

Reliability analyses indicate that selfie-takers show a reasonable degree of internal consistency in their selfie pose selections: Cronbach’s α = 0.720 (female participants: Cronbach’s α = 0.712; male participants: Cronbach’s α = 0.725). Overall, there were more left (*N* = 779) and fewer midline (*N* = 535) selfies than would be expected by chance (expected *N* = 666.7; right cheek pose *N* = 686; please note that “left” and “right” always refer to the selfie-takers’ anatomical cheek offered to the camera or mirror). Comparing only left and right cheek poses indicates that selfie takers show a left cheek bias: 53.17% left cheek selfies; 46.83% right cheek selfies. **Figure 1** illustrates the percentages of left, right, and midline selfies for male and female selfie-takers.

The number of left, right, and midline selfie poses for each participant were calculated to examine intra-individual consistency in pose orientation across 10 selfies. Given the three posing options (left cheek, midline, and right cheek pose), five or more selfies in one pose orientation was classed as an overall bias toward that pose orientation, being higher than the frequency expected by chance (3.33/10). Participants whose selfie poses were evenly distributed, with no more than four selfies in any of the three pose categories, were classified as having no overall bias. Similarly, three participants who had five selfies in each of two pose categories, and no selfies in the third category, were included in the no overall bias group. Only 16 participants showed no overall bias. The vast majority of participants (*N* = 184) showed an overall bias across their 10 single-subject selfies favoring one of the three pose orientations: left cheek bias mean 6.61 left cheek poses/10 (*SD* = 1.37); midline bias mean 6.54 midline poses/10 (*SD* = 1.59); right cheek bias mean 6.70 right cheek poses/10 (*SD* = 1.50). Of the participants who showed a very strong bias, with 8 or more out of their 10 selfies in one pose orientation, 20 had a left cheek bias (mean 8.50 left cheek poses/10, *SD* = 0.61), 9 had a midline bias (mean 8.89 midline poses/10, *SD* = 0.93), and 14 had a right cheek bias (mean 8.86 right cheek poses/10, *SD* = 0.77).

**FIGURE 2:**
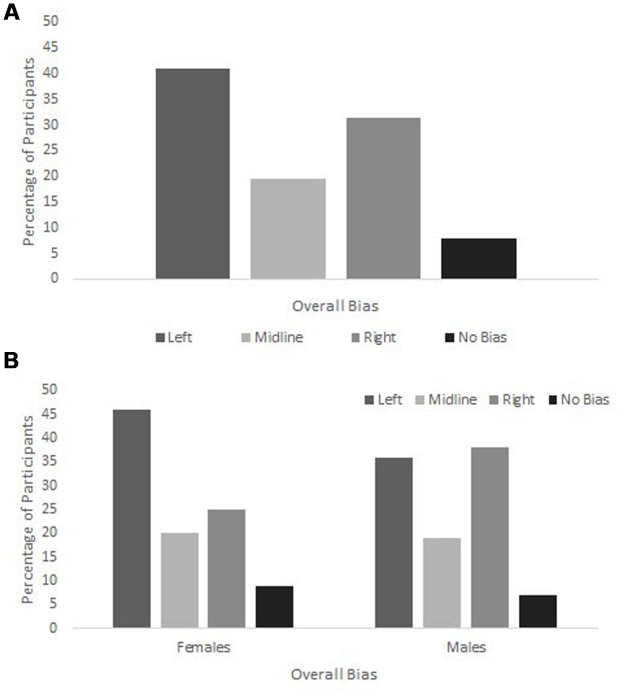
**Percentages of overall left cheek, midline, right cheek, and no bias across 10 selfies for** **(A)** all participants, and **(B)** as a function of selfie-taker’s sex (female, male). Please note that ‘cheek’ refers to the anatomical cheek offered to the camera or mirror.

The observed frequencies of overall left, midline, and right cheek biases were tested against a null model that assumes that the three posing categories (left, midline, right) are equally probable. The model thus assumes the frequencies expected by chance: 0.333 for each of the three pose categories (as 16/200 participants showed no overall bias, the null model’s chance *N* = 184/3 = 61.33). Results revealed that the number of participants exhibiting an overall left cheek bias was higher (*N* = 82), and an overall midline bias was lower (*N* = 39), than would be expected by chance [χ^2^(2) = 15.141, *p* = 0.001; please refer to **Figure 2**]; the frequency of an overall right cheek bias was consistent with that anticipated by chance (*N* = 63). Comparison of overall biases for females and males indicates that frequencies for both groups differed from those anticipated by chance [females: χ^2^(2) = 12.549, *p* = 0.002; males: χ^2^(2) = 7.032, *p* = 0.030; please refer to **Figure 2**]. However, it could be argued that a null model with equiprobable frequencies for overall left, midline, and right cheek biases does not necessarily reflect the probabilities of selfie poses, the range of rotation angles included in the ‘midline’ category being more restricted than those of the ‘left’ and ‘right’ categories.

A pair of repeated measures logistic regressions were thus conducted. In the first analysis, midline poses were compared to left and right poses to assess the factors that determine whether people favor midline over asymmetric selfie poses. Repeated measures logistic regression modeled the relationship between portrait orientation (midline, left/right) and the predictor variables sex (female, male) and selfie type (normal mirror), controlling for the repeated measure of selfie-taker identity. Results indicated that neither sex [χ^2^(1) = 0.820, *p* = 0.820] nor selfie type [χ^2^(1) = 1.876, *p* = 0.171] influenced selfie pose orientation. The interaction between sex and selfie type was similarly non-significant [χ^2^(1) = 0.297, *p* = 0.586].

**FIGURE 3:**
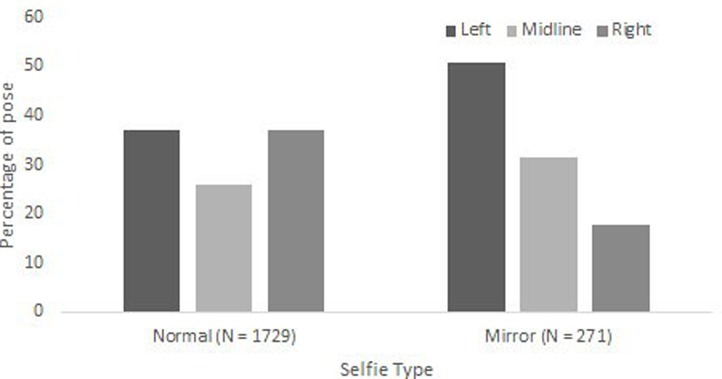
**Percentages of left, right, and midline selfie poses as a function of selfie type (normal, mirror).** Please note that ‘cheek’ refers to the anatomical cheek offered to the camera or mirror.

A second repeated measures logistic regression was then performed to examine the factors that influence preferences for asymmetric selfie poses only; midline poses were removed from the analysis. The model assessed the relationship between portrait orientation (left, right) and the predictor variables sex (female, male) and selfie type (normal mirror), controlling for the repeated measure of selfie-taker identity. Results indicated that whilst sex [χ^2^(1) = 1.368, *p* = 0.242] did not predict pose orientation, selfie type proved a highly significant predictor [χ^2^(1) = 14.061, *p* = 0.000]. For normal selfies, left (*N* = 641) and right (*N* = 638) cheek poses were similarly frequent, whereas for mirror selfies, left cheek (*N* = 138) poses were more frequent than right cheek poses (*N* = 48; please refer to **Figure 3**). The interaction between sex and selfie type was not significant [χ^2^(1) = 0.088, *p* = 0.767].

## Discussion

Research has established that the left cheek bias for painted (e.g., McManus and Humphrey, 1973) and photographic (e.g., LaBar, 1973) portraits of others is similarly evident in selfies (e.g., Bruno and Bertamini, 2013). As previous investigations of selfie posing biases have sampled a single selfie from multiple different participants, whether individual selfie-takers consistently favor their ‘best side’ when taking selfies, and whether a left cheek bias is evident intra-individually, was not known. The present study reveals that the vast majority of selfie-takers show an overall bias, repeatedly favoring one pose orientation (left cheek, midline, or right cheek). Critically, more participants showed a left cheek bias within their own catalog of selfies than would be expected by chance; the left cheek bias previously observed across individuals is present intra-individually. Analysis comparing left and right cheek selfies and controlling the repeated measure of selfie-takers’ identity indicated that while sex did not predict selfie pose orientation, the left cheek bias was stronger for mirror, than normal, selfies. Overall, these results are in line with previous reports in confirming a left cheek bias for selfies. Moreover, they indicate that selfie-takers tend to consistently adopt one pose orientation, presumably favoring their best side.

Examination of the 10 most recent selfies participants uploaded to Instagram confirms that individual selfie-takers consistently prefer one pose orientation. Ninety-two percent of the sample showed an overall posing bias, with 41% favoring their left cheek, 31.5% preferring their right cheek, and 19.5% repeatedly posting midline selfies. Given that only 8% of selfie-takers showed no overall bias, the tendency to repeatedly adopt a preferred pose appears to be the norm for selfie takers. Importantly, the number of participants showing an overall left cheek bias was significantly higher than expected by chance, indicating an intra-individual left cheek bias for selfies that complements the previously reported left cheek bias across individuals (Bruno and Bertamini, 2013; Bruno et al., 2014, 2015, in press). The greater frequency of an overall left cheek bias was similarly consistent at the upper end of the bias spectrum. Of the 43 participants who showed a very strong bias toward one pose in their selfie corpora (8 or more out of 10 selfies in one pose orientation), 46.5% had 8 or more left cheek selfies, 32.6% had 8 or more right cheek selfies, and 20.9% had 8 or more midline selfies. Given that selfies show the world one’s subjective self-image (Souza et al., 2015), the greater than expected proportion of participants showing an overall left cheek bias suggests that selfie-takers intuitively favor the more emotionally expressive self-representation communicated in left cheek and midline, rather than right cheek, poses (e.g., Nicholls et al., 2002).

The intra-individual consistency in pose choice (α = 0.72), and consistency in the magnitude of the intra-individual bias (overall left cheek bias: mean 6.61 left cheek selfies/10 selfies; overall midline bias: 6.54 midline selfies/10 selfies; overall right cheek bias: 6.70 right cheek selfies/10 right cheek selfies), suggests that whilst selfie-takers repeatedly favor a preferred pose, they occasionally switch pose orientations. Whether this is done consciously, to avoid monotony, or unconsciously, potentially reflecting state-based differences in selfie-takers’ mood, is presently unknown. Moreover, whether selfie posing biases influence the number of ‘likes’ the selfies garner (‘likes’ being currency in social media, with selfies generally capturing 1.1–3.2 times more likes and comments than other types of images posted on Instagram, Souza et al., 2015) remains an open question.

Across the total sample of 2000 selfies, midline selfies were less frequent (26.75%) than either left cheek (38.95%) or right cheek (34.30%) selfies. This tendency to favor lateral, rather than head-on, selfie poses has been repeatedly reported in previous lab-based (Bruno and Bertamini, 2013; Bruno et al., in press) and real world (Bruno et al., 2015) investigations. Whilst research indicates that midline portrait poses are perceived as being just as emotionally expressive as left cheek poses (Nicholls et al., 2002), Lindell (2017) suggested that midline poses are less frequently adopted for a simple reason: they appear less flattering (e.g., driver’s license, passport photo). Tips for posing for the “perfect portrait” and the “perfect selfie” regularly include avoiding facing the camera head on in a midline pose, unless one is aiming to look bigger; instead, adopting a 3/4 or 2/3 turn toward the camera is encouraged because it introduces more angles, highlights the cheekbones, and makes the subject of the photo appear slimmer (e.g., Manning, 2011; Olsen, 2012). As the subjects of selfies simultaneously serve as their own photographers, they are in the position to take multiple images and try out multiple poses to find the best (and potentially the most flattering to one’s self-concept, Döring et al., 2016), before uploading the chosen selfie to social media. This may explain the smaller proportion of midline selfies observed in the present investigation, however, research examining the relationship between selfie pose orientation and perceived emotionality, attractiveness, masculinity, or femininity, is clearly needed to assess this speculation.

Controlling for the repeated measure of selfie-taker identity, analysis comparing left and right cheek poses indicated that selfie type (normal, mirror) predicted pose orientation, revealing a stronger left cheek bias for mirror than normal selfies. Bruno et al. (2015) found a similar pattern in their examination of the SelfieCity database (selfies drawn from Instagram), arguing that this reflects differences in the mechanics of selfie taking. Most selfie-takers capture their selfies by holding the smartphone in their dominant hand (Lindell, 2017). In mirror selfies the smartphone is typically held centrally, near the body (Bruno et al., 2015), and when held with the right hand, may be placed slightly right of midline. Thus presuming that the majority of the sample is right-handed (a reasonable presumption, given the near-universal preference for the right hand, Corballis, 2014), this makes left cheek poses easier to adopt in mirror than normal/standard selfies (Bruno et al., 2015), potentially resulting in the higher proportion of left cheek poses observed. This argument is necessarily speculative because although motor biases (including handedness and hand used to capture the selfie) do not influence posing biases for normal selfies (Lindell, 2017), research has yet to investigate the influence of motor biases on mirror selfie pose orientation.

The absent effect of participant sex on pose orientation is also consistent with previous selfie investigations (Bruno et al., 2015; Lindell, 2017). Though inspection of the present data suggests a stronger left cheek bias for females than males (see **Figure 2**), sex was not a significant predictor of pose in either of the logistic regression analyses. Both Bruno et al. (2015) and Lindell (2017) similarly found that selfie pose orientations were not affected by participants’ sex (sex was not examined as a factor in Bruno and Bertamini, 2013; Bruno et al., 2014, in press), suggesting that posing biases in selfies deviate from those observed for portraits of others. The stronger left cheek bias for females typically reported for portraits of others (e.g., McManus and Humphrey, 1973) is compatible with an emotion lateralization account of the left cheek bias (see Lindell, 2013b, for review). As females are more emotionally expressive (e.g., Kring et al., 1994), they are more likely to intuitively pose offering the more emotionally expressive left cheek.

The fact that sex did influence selfie pose orientation in the present study or previous investigations (e.g., Bruno et al., 2015; Lindell, 2017) could reflect an effect of the genre: males may feel more comfortable expressing emotion when capturing their own image in a selfie than when posing for another, especially when encouraged to pose “as you really are” [as instructed in Lindell’s (2017) investigation]. Equally, the lack of a sex effect for selfies could reflect changes in contemporary gender expectations and the characteristics of the population who upload selfies to Instagram for public consumption (both the present study and Bruno et al., 2015, used Instagram-based selfie samples). Sorokowska et al. (2016) found that higher levels of exhibitionism and extraversion characterize people who post selfies more frequently, irrespective of sex. One may speculate that the (typically young) males uploading selfies to Instagram are less constrained by social mores, and thus are more willing to express emotion, than males posing for professional portraits in previous generations (e.g., McManus and Humphrey, 1973). In keeping with this argument, recent research assessing emotional expressivity in college students found no difference between males’ and females’ levels of emotional expressivity (e.g., Lü and Wang, 2012), thus the lack of a sex difference in posing biases in the present study remains consistent with the emotion lateralization-based account of the left cheek bias. Further research examining whether males’ and females’ personality and emotional expressivity predict selfie pose orientations is needed to confirm this speculation.

The selfies sampled in the present study were explicitly identified as selfies (#selfie) and uploaded to Instagram by the selfie-takers for public viewing. Thus, like Bruno et al.’s (2015) selfie investigation, the present study has excellent ecological validity. It is therefore encouraging that the results observed in laboratory-based investigations of selfie-taking (e.g., Bruno et al., 2014, in press) match those found using these real-world samples. This consistency in findings is a clear indicator of the robustness of the left cheek bias for portraits, being evident across painted (e.g., McManus and Humphrey, 1973) and photographic (e.g., LaBar, 1973) portraits of others, and in both lab-based (e.g., Bruno et al., in press) and real-world (e.g., Bruno et al., 2015) samples of selfies. The present study confirms that the left cheek bias also manifests within an individual’s selfie corpus, with more people consistently adopting a left cheek pose than either of the other posing options. Given the effort exerted in taking and retaking selfies to find the perfect angle (Hess, 2015), with young women discarding six selfies for each selfie uploaded (Strick, 2015), such findings imply that there is something very special about the left cheek. Previous research indicates that left cheek poses are perceived as more emotionally open and expressive than right cheek poses (Nicholls et al., 2002), and we know that selfie-takers perceive themselves as more attractive and likable in their selfies than in other photographs (Re et al., 2016). Whether left cheek selfies induce a more positive impression of the selfie-taker in other perceivers remains to be determined.

## Author Contributions

AL designed the study, analyzed and interpreted the data, and wrote the paper.

## Conflict of Interest Statement

The author declares that the research was conducted in the absence of any commercial or financial relationships that could be construed as a potential conflict of interest.

## Footnotes

1 http://www.instagram.com/press
